# Flea Diversity as an Element for Persistence of Plague Bacteria in an East African Plague Focus

**DOI:** 10.1371/journal.pone.0035598

**Published:** 2012-04-18

**Authors:** Rebecca J. Eisen, Jeff N. Borchert, Joseph T. Mpanga, Linda A. Atiku, Katherine MacMillan, Karen A. Boegler, John A. Montenieri, Andrew Monaghan, Kenneth L. Gage

**Affiliations:** 1 Division of Vector Borne Diseases, Centers for Disease Control and Prevention, Fort Collins, Colorado, United States of America; 2 Uganda Virus Research Institute, Entebbe, Uganda; 3 National Center for Atmospheric Research, Boulder, Colorado, United States of America; Global Viral Forecasting Initiative, United States of America

## Abstract

Plague is a flea-borne rodent-associated zoonotic disease that is caused by *Yersinia pestis* and characterized by long quiescent periods punctuated by rapidly spreading epidemics and epizootics. How plague bacteria persist during inter-epizootic periods is poorly understood, yet is important for predicting when and where epizootics are likely to occur and for designing interventions aimed at local elimination of the pathogen. Existing hypotheses of how *Y. pestis* is maintained within plague foci typically center on host abundance or diversity, but little attention has been paid to the importance of flea diversity in enzootic maintenance. Our study compares host and flea abundance and diversity along an elevation gradient that spans from low elevation sites outside of a plague focus in the West Nile region of Uganda (∼725–1160 m) to higher elevation sites within the focus (∼1380–1630 m). Based on a year of sampling, we showed that host abundance and diversity, as well as total flea abundance on hosts was similar between sites inside compared with outside the plague focus. By contrast, flea diversity was significantly higher inside the focus than outside. Our study highlights the importance of considering flea diversity in models of *Y. pestis* persistence.

## Introduction

Plague is a flea-borne rodent-associated zoonotic disease that is caused by *Yersinia pestis* and characterized by long quiescent periods punctuated by rapidly spreading epidemics and epizootics. *Yersinia pestis* is notorious for causing three major human pandemics that killed millions. The magnitude of these pandemics has been credited for changing economic and political history and for notable improvements in public health [1], [2]. In modern times, advances in diagnostics coupled with access to appropriate antibiotic therapy have resulted in a reduction in plague-related mortality rates; improved sanitation has limited the scale of epidemics to focal outbreaks [3]. Nonetheless, plague remains a standard against which modern scourges are compared and the recrudescence of plague can cause major disruptions to social and economic infrastructure at local scales that can have global ripple effects [4]. Defining the geographic foci in which *Y. pestis* persists during inter-epizootic periods and understanding the mechanisms of persistence are critical for designing interventions aimed at locally eliminating the pathogen and for anticipating where future epizootics are most likely to occur.

Plague foci represent areas where *Y. pestis* has persisted over long durations of time spanning multiple epidemics or epizootics that are often disrupted by periods of low and often undetectable transmission. These foci are commonly characterized based on landscape (e.g., elevation, vegetation) and climatic variables [3], [5], [6], [7], [8], [9], [10], [11] or types of hosts [12]. In the 20^th^ century, primarily in central Asia, the notion that host ecology was paramount to *Y. pestis* persistence was so engrained that foci were often named for the dominant host species in that locality[13], [14].

Within plague foci, the timing of epizootics is often predicted based on temperature and precipitation [15]. Why weather and climate are often predictive of the temporal and spatial distributions of *Y. pestis*, respectively, is poorly understood. Temperature and precipitation may affect the distribution and abundance of key hosts and vectors involved in *Y. pestis* transmission [16], [17], [18], [19], [20]. Alternatively, host and flea communities could remain stable and instead temperature could regulate the ability of *Y. pestis* to persist in, or be transmitted by, fleas.

Prior to the last pandemic, the majority of human plague cases was reported from temperate foci where seasonality is clearly defined and there is marked variation in monthly temperatures [3]. However, in recent decades, most human plague cases have been reported from tropical foci in east Africa and Madagascar where temperature extremes are less pronounced than in temperate foci [3]. Our study centers on the West Nile region of Uganda, which represents an epidemiological focus for plague in this country. Recent studies have identified landscape level differences between localities in the West Nile region that have reported human plague cases and those that have not [6], [11], [21]. Consistent with plague foci in North America and other parts of east Africa, the West Nile focus was defined based on elevation, wetness, and land use. However, it remains unclear how landscape level differences relate to mechanisms that account for differences in plague risk. Here, we sought to determine if abundance or diversity of hosts and fleas differed among villages inside versus outside of this recently delineated human plague focus in the West Nile region of Uganda. Our findings highlight the importance of flea diversity in *Y. pestis* maintenance or transmission to humans within this east African plague focus.

## Materials and Methods

### Description of the study site

**Figure 1:**
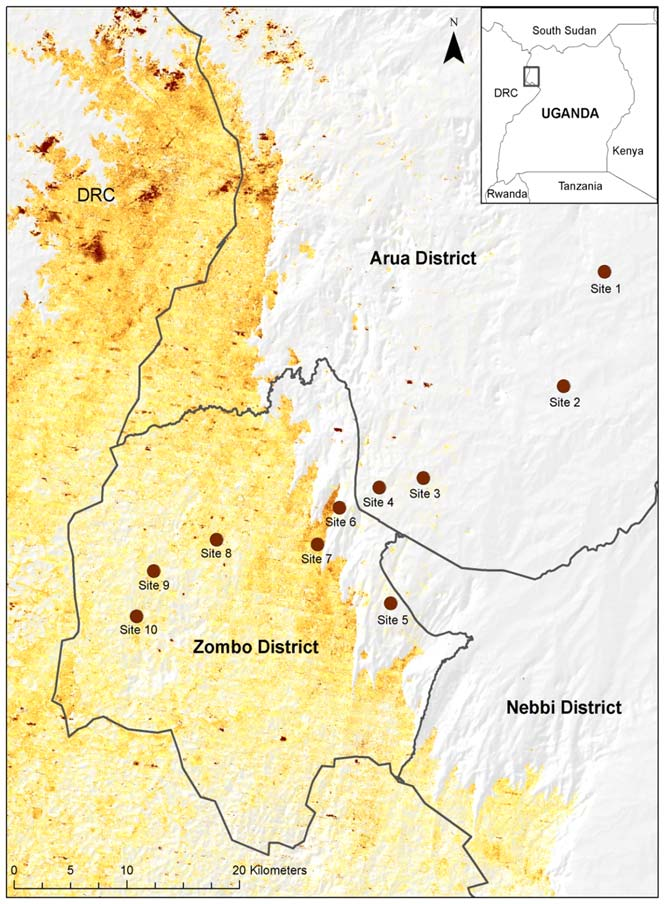
Location of study sites in relation to areas of elevated risk for plague (color gradient) [**6**]. Inset shows location of the area of interest within Uganda. Sites 1–6 are situated below 1300 m and sites 7–10 are above this elevation threshold.

Our sampling was focused in Arua and Zombo districts in the plague-endemic West Nile Region of northwestern Uganda. The majority of human plague cases in these districts have been reported from localities situated above the Rift Valley escarpment, which roughly bisects our study site [6], [11]. We established 10 small mammal sampling sites that were evenly spaced with respect to elevation along a transect spanning from approximately 725 (site 1) to 1630 m (site 10) in elevation (average difference in elevation between neighboring sites: 99 m, range: 73–127 m). Half of the sites were situated in areas identified by our previous risk model as posing an elevated risk for plague (sites 6–10) [6] and four of the highest elevation sites (sites 7–10) were above the 1300 m elevation threshold that was previously identified as delineating differences between areas of low or elevated plague risk (Figure 1) [6], [11]. Our delineation of areas that pose an elevated risk was based on approximately 10 years of epidemiological surveillance data collected from clinics above and below the 1300 m elevation threshold [6], [11]. Animal-based surveillance for plague activity is not routinely conducted in Uganda, in part due to the cost but also because seropositivity and culture recovery efforts are extremely low, especially during inter-epizootic periods. Thus, although humans are incidental hosts of *Y. pestis*, the epidemiological dataset provided the best available estimate of plague activity in the region. It is possible that *Y. pestis* may circulate in small mammal communities outside of the identified epidemiological focus. However, surveys designed to assess differences in human demographics or behaviors that we believed to be important risk factors for plague (e.g., household size, food storage and agricultural practices and vector and rodent control methods) revealed no obvious differences between high (>1300 m) and low elevation sites. Thus, lack of human infections at low elevation sites is likely attributable to either a lack of bridging vectors between zoonotic cycles and human hosts, or lack of *Y. pestis*. Sites located above 1300 m were generally wetter (median cumulative annual rainfall [range]: 1644.8 mm [1500.2–1889.2 mm]) and cooler (median average maximum monthly temperature [range]): 26.1°C [25.3–27.0°C]) than sites below this elevation (1080 mm [864.2–1483.3 mm]; 30.6°C [28.3–32.7°C]). Site-specific rainfall and temperature data were derived from high-resolution atmospheric model simulations for 1999–2008, described below.

### Description of small mammal and flea collection

Small mammals and their associated fleas were collected from 40 homesteads situated within the 10 trap sites described above. Timing of the five collections coincided primarily with the rainy seasons. Collections were conducted in February–March 2010 and 2011, which mark the end of the dry season and the start of the secondary rainy season, and October 2010 and November 2010, which represents the peak of the primary rainy season, and April 2011, which falls in the middle of the secondary rain season.

**Figure 2:**
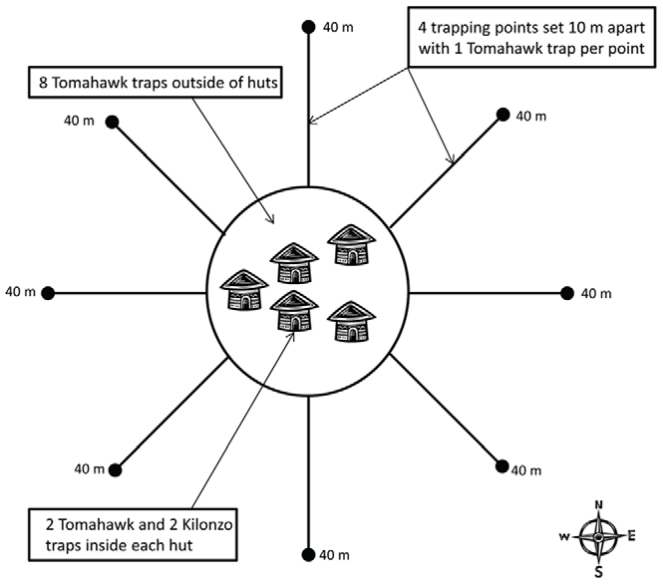
Schematic of sampling design for a single homestead within a village.

For each homestead, which is comprised of a small cluster of traditional homes constructed of mud waddle and thatch roofing (mode: 5 huts, range 2–6 huts per homestead; median numbers of huts included per elevation were similar), 2 Tomahawk and 2 Sherman traps were set inside of each hut, 8 Tomahawk and 8 Sherman traps were set in the family compound outside of the huts, and 64 traps comprised of 32 Tomahawk and 32 Sherman traps were set in the peridomestic setting extending 40 m away from the huts. One Tomahawk and one Sherman trap was placed per trap station and trap stations were set 10 m apart radiating from the homestead in the 4 cardinal and 4 inter-cardinal directions (figure 2). Traps were baited with a mixture of 1/3 corn, 1/3 ground nuts, and 1/3 dried fish. For each trap session, trapping was conducted for two consecutive nights with rodents retrieved each morning and traps reset at dusk. We acknowledge that our sampling is biased towards nocturnally active small mammals and abundances of diurnal rodents, such as *Arvicanthis niloticus* may be underrepresented. Upon capture, small mammals were anesthetized using an inhalation anesthetic (halothane). Sedated animals were measured, weighed, identified to genus or species and combed for fleas. For host species that could not be identified to species because of a lack of morphological differences that were detectable in the field, individuals were identified to genus (e.g., *Crocidura spp.*, *Mastomys spp.* and *Praomys spp.*). Collected fleas were preserved in 70% ethanol for later identified to species following published taxonomic keys [22], [23], [24], [25]. All animal procedures described in the study were approved by the Centers for Disease Control and Prevention Division of Vector Borne Diseases Institutional Animal Care and Use Committee (protocol 09-023).

Host-seeking fleas were collected from each of the same huts described above for 2 consecutive nights during each small mammal trapping session by placing 1 modified Kilonzo pan trap [26], [27] on the floor in locations deemed to be the most protected from animals that are frequently in the huts (e.g., chickens, goats, dogs) and were placed away from areas where residents slept. The pan traps measured 20.0 cm in diameter and 3.0 cm in depth. A flashlight was suspended above the pans in a solution of 2% saline with Tween 80 (5 drops of Tween 80 added to 1.0 cm to 1.5 cm in depth of saline). Petroleum jelly was applied to the rim of the pan to prevent fleas from escaping. After each of the 2 nights, fleas were collected from the pans and stored in 70% ethanol until species identification could be performed.

### Climate variables

Meteorological observation networks are sparse in sub-Saharan Africa, and no observation stations were established within our study area. As a result, to assess the relationship between flea diversity and climatic variables, we used ten-year averages (1999–2008) for cumulative monthly and annual precipitation, minimum temperature during the coolest month (June) and maximum temperature during the hottest month (February) of the year derived from a suite of atmospheric simulations performed specifically for the West Nile region with the Weather Research and Forecasting Model (WRF) [28]. WRF is a fully compressible conservative-form nonhydrostatic atmospheric model suitable for both research and weather prediction applications; it has demonstrated ability for resolving small-scale phenomena and clouds [29]. Monaghan et al. [28] demonstrated that the resulting WRF-based climate dataset better resolves the spatial variability and annual cycle of temperature, humidity, and rainfall in West Nile compared to satellite-based and *in situ* records, and therefore is suitable for use in this study.

### Data analysis

Relative host abundance, measured as the number of hosts per trapping effort, was determined for each elevation point by dividing the numbers of hosts of a particular genus or species collected within an elevation point (e.g., sites 1–10 shown in figure 1) by the number of trap nights per site. Host diversity was estimated for each sampling site using Simpson's Index of Diversity [30], described as:
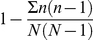
where n is the total number of hosts of a particular species or genus and N is the total number of hosts of all species. Flea diversity, based on on-host fleas, was estimated using the same equation. Hosts and fleas used in these calculations correspond with those listed in Table 1. The Simpson's index of diversity ranges from 0 to 1 with the greater value indicating greater diversity within the sample. Median numbers of hosts per trap, total number of fleas collected from host, fleas per host, total numbers of host-seeking fleas, and diversity indices were compared between sites above or below 1300 m elevation using Wilcoxon rank sum tests with chi square approximations.

**Table 1:** Flea infestations of small mammals collected from sites 1–10, February 2010–April 2011.

		Total no. fleas collected per host (no. fleas/host examined)												
Host species	No. hosts	Xc	Xb	Cc	Xn	Dl	St	Eg	Cb	Cf	Dlo	La	Tp	Total
*R. rattus*	546	220 (0.403)	113 (0.207)	0 (0.000)	3 (0.005)	9 (0.016)	4 (0.007)	96 (0.176)	2 (0.004)	2 (0.004)	0 (0.000)	1 (0.002)	1 (0.002)	460 (0.842)
*A. niloticus*	310	286 (0.923)	49 (0.158)	127 (0.410)	6 (0.019)	118 (0.381)	0 (0.000)	0 (0.000)	8 (0.026)	1 (0.003)	1 (0.003)	0 (0.000)	0 (0.000)	596 (1.923)
*Crocidura spp.*	178	123 (0.691)	6 (0.034)	7 (0.039)	0 (0.000)	7 (0.039)	104 (0.584)	0 (0.000)	0 (0.000)	0 (0.000)	0 (0.000)	0 (0.000)	0 (0.000)	247 (1.388)
*Mastomys spp.*	130	68 (0.523)	21 (0.162)	6 (0.046)	2 (0.015)	1 (0.008)	7 (0.054)	0 (0.000)	0 (0.000)	0 (0.000)	0 (0.000)	0 (0.000)	0 (0.000)	105 (0.808)
*M. minutoides*	88	0 (0.000)	0 (0.000)	0 (0.000)	0 (0.000)	0 (0.000)	4 (0.045)	0 (0.000)	0 (0.000)	0 (0.000)	0 (0.000)	0 (0.000)	0 (0.000)	4 (0.045)
*T. emini*	79	3 (0.057)	0 (0.000)	0 (0.000)	68 (0.86)	1 (0.019)	0 (0.000)	0 (0.000)	0 (0.000)	0 (0.000)	0 (0.000)	0 (0.000)	0 (0.000)	73 (0.92)
*T. valida*	74	8 (0.108)	0 (0.000)	0 (0.000)	82 (1.108)	2 (0.027)	0 (0.000)	1 (0.014)	0 (0.000)	0 (0.000)	0 (0.000)	0 (0.000)	0 (0.000)	93 (1.257)
*A. hindei*	40	80 (2.000)	0 (0.000)	0 (0.000)	0 (0.000)	0 (0.000)	0 (0.000)	0 (0.000)	0 (0.000)	0 (0.000)	0 (0.000)	0 (0.000)	0 (0.000)	80 (2.000)
*L. striatus*	24	0 (0.000)	0 (0.000)	6 (0.039)	0 (0.000)	3 (0.0125)	0 (0.000)	0 (0.000)	0 (0.000)	0 (0.000)	0 (0.000)	0 (0.000)	0 (0.000)	11 (0.458)
*L. flavopunctatus*	20	0 (0.000)	2 (0.100)	17 (0.850)	0 (0.000)	2 (0.1)	8 (0.400)	0 (0.000)	0 (0.000)	0 (0.000)	0 (0.000)	0 (0.000)	0 (0.000)	29 (1.450)
*Praomys spp.*	6	0 (0.000)	0 (0.000)	0 (0.000)	0 (0.000)	0 (0.000)	1 (0.167)	0 (0.000)	0 (0.000)	0 (0.000)	0 (0.000)	0 (0.000)	0 (0.000)	1 (0.167)
*C. gambianus*	4	0 (0.000)	0 (0.000)	0 (0.000)	0 (0.000)	0 (0.000)	0 (0.000)	0 (0.000)	0 (0.000)	0 (0.000)	0 (0.000)	0 (0.000)	0 (0.000)	0 (0.000)
*Thamnomys spp.*	3	6 (2.000)	0 (0.000)	0 (0.000)	0 (0.000)	0 (0.000)	0 (0.000)	0 (0.000)	0 (0.000)	0 (0.000)	0 (0.000)	0 (0.000)	0 (0.000)	6 (2.000)
*Lophuromys sikapusi*	1	0 (0.000)	0 (0.000)	1 (1.000)	0 (0.000)	1 (1.000)	0 (0.000)	0 (0.000)	0 (0.000)	0 (0.000)	0 (0.000)	0 (0.000)	0 (0.000)	2 (2.000)
Total	1503	797 (0.530)	191 (0.127)	173 (0.115)	161 (0.107)	144 (0.095)	128 (0.085)	97 (0.065)	10 (0.007)	3 (0.002)	1 (0.001)	1 (0.001)	1 (0.001)	1707 (1.135)

Hosts and fleas are listed in descending order of abundance.

Fleas: Cf: *Ctenocephalides felis*; Cc: *Ctenophthalmus cabirus*; Cb: *Cetonphthalmus bacopus*; Dl: *Dinopsyllus lypusus*; Dlo: *Dinopsyllus longifrons*: Eg: *Echinophaga gallinacea*; La: *Leptopsyllus aethiopicus*; St: *Stivalius torvus*; Tp: *Tunga penetrans*; Xb: *Xenopsylla brasiliensis*; Xc: *Xenopsylla cheopis*; Xn: *Xenopsylla nubica*.

Hosts: *R. rattus*: *Rattus rattus*; *A. niloticus*: Arvicanthis niloticus; *M. minutoides*: *Mus minutoides; T. emini: Taterillus emini; T. valida: Tatera valida; A. hindei: Aethomys hindei: L. striatus: Lemniscomys striatus; L. flavopunctatus: Lophuromys flavopunctatus; C. gambianus: Cricetomys gambianus; L. sikapusi: Lophuromys sikapusi*.

The relationship between elevation and flea diversity was explored using linear regression. To evaluate the association between flea diversity and rainfall, forward stepwise regression was used to screen average monthly site-specific values of rainfall. Rainfall in this region follows a bimodal distribution with the reliable heavy rain falling from August through November during the primary rainy season. December through February marks the dry season, which is followed by the less reliable and often lighter rainy season from March–June. June and July are characterized as the interval season with variable amounts of rain [28]. Spatial variation in rainfall between sites for any particular month can be considerable [21], therefore we used a forward stepwise screening process that included each average monthly value for rainfall to identify the best statistical fit to the flea diversity data. Regardless of the month, temperatures consistently decreased with increasing elevation and temperature showed only a single peak with highest temperatures observed in February and the lowest temperatures in June. Therefore, average minimum temperature in June (lowest temperature during the coolest month) and average maximum temperature for February (highest temperature for the warmest month) were included in models of flea diversity. These temperature extremes were selected because flea survival is often temperature dependent and average values may have obscured the true range in temperature experienced by the fleas. For both the rainfall and the temperature models, the best model was selected based on comparisons of the corrected Akaike Information Criterion. Because only 10 sites were included in the model, we restricted the number of predictive variables to one.

## Results

### Description of host and flea diversity along the elevation gradient

**Figure 3:**
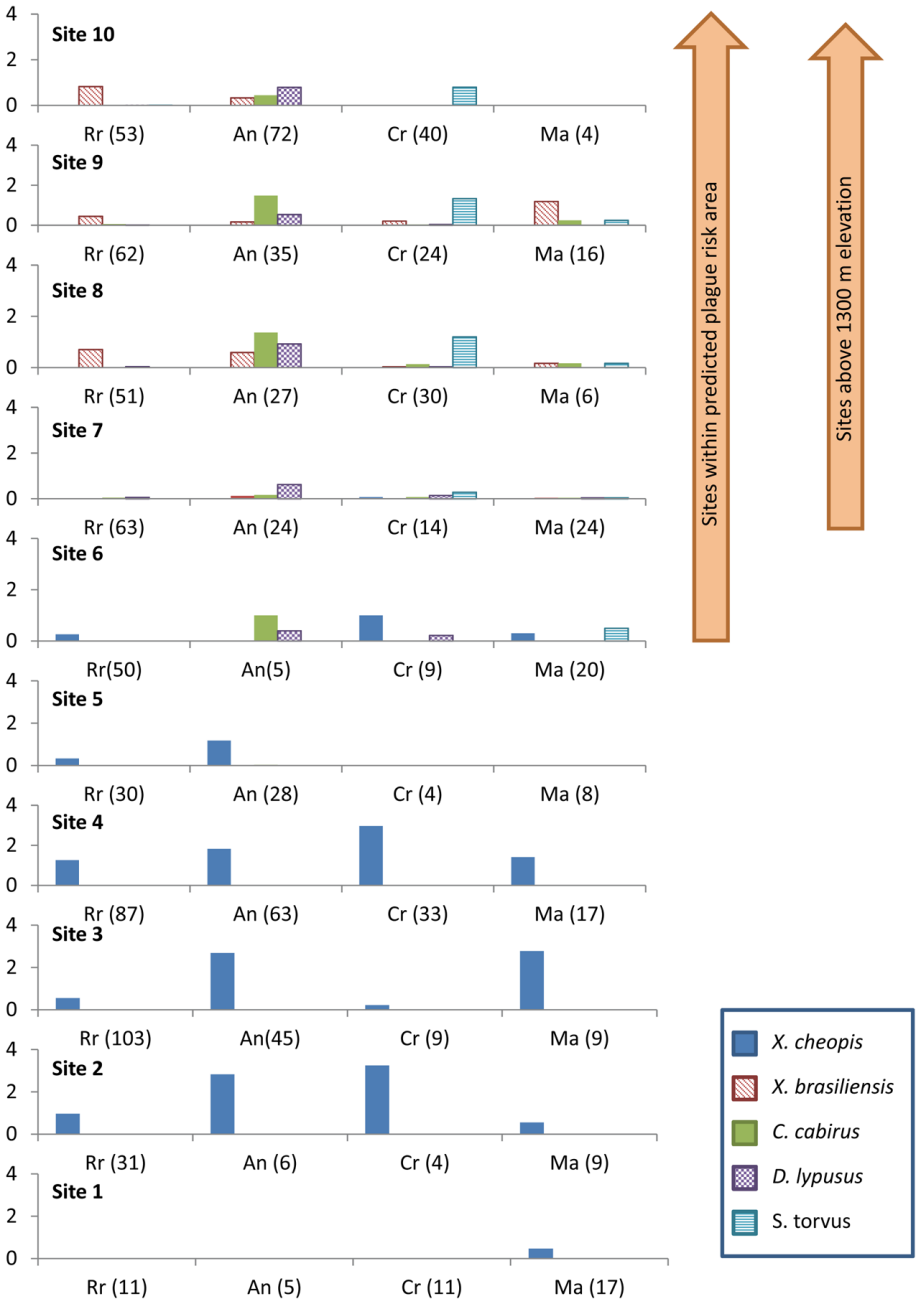
Flea infestations of key hosts and *Y. pestis* vector species among sampling sites. *Rattus rattus*, *Arvicanthis niloticus*, *Crocidura spp.* and *Mastomys spp.* are indicated as Rr, An, Cr, and Ma, respectively. Numbers of hosts examined per site is given in parentheses.

During a total of 7,832 trap nights spanning from 22 February 2010 to 10 April 2011 and run within 40 homesteads at 10 elevation points, a total of 1,503 small mammals comprised of at least 14 species were captured. Together, the four most commonly collected hosts (*Rattus rattus*, *Arvicanthis niloticus*, *Crocidura spp.* and *Mastomys spp.*) accounted for 77% of all hosts examined (Table 1). From all hosts examined, a total of 1,707 fleas comprised of 12 species were collected. Five species were extremely rare, each representing less than 1% of the sample (Table 1). Subsequent analyses in this sub-section focus on the five species of fleas that were commonly associated with the four most abundant host species identified above: *Xenopsylla cheopis*, *X. brasiliensis*, *Ctenophthalmus cabirus*, and *Dynopsyllus lypusus*, and *Stivalius torvus*. Although *X. nubica* was more abundant than *D. lypusus* and *S. torvus*, they were collected primarily from gerbils (belonging to the genus *Tatera* or *Taterillus*), which represented only 10% of all hosts collected (Table 1). *Xenopsylla cheopis* and *X. brasiliensis* were collected commonly from each of the four key host species, but *X. cheopis* was rare above 1300 m (only 1 *X. cheopis* was recovered above 1300 m) and *X. brasiliensis* was absent on sampled hosts below this elevation threshold (Figure 3). By contrast, with the exception of a single *C. cabirus* collected at site 5, *C. cabirus*, *D. lypusus*, and *S. torvus* were not observed in sites 1–5, were observed in low numbers at site 6 (n = 4 *D. lypusus* and 1 each of *C. cabirus* and *S. torvus*) and were common at sites 7–10 (figure 3). It is noteworthy that site 6, although lower than 1300 m elevation, marks the first site to appear within the predicted elevated risk area (figure 1).

During approximately 1,790 Kilonzo trap nights, a total of 836 host-seeking fleas were collected. *Ctenocephalides felis* and *Echinophaga gallinacea* represented 61% (n = 510) and 37% (n = 311) of the captures, respectively. Other species captured included *Tunga penetrans* (n = 2), *X. brasiliensis* (n = 1) and *X. cheopis* (n = 12). The total numbers of fleas collected, and total numbers of the nearly cosmopolitan flea species, *C. felis* and *E. gallinacea*, were similar between sites located above compared with below 1300 m elevation.

### Comparisons of host abundance, flea infestation, and host and flea diversity between sites situated above versus below 1300 m elevation

**Figure 4:**
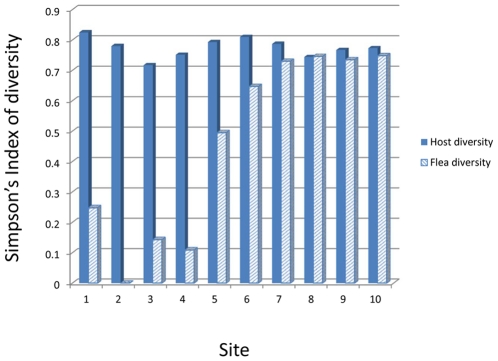
Comparison of host and flea diversity among sampling sites. Host diversity indices were similar between sites above (7–10) and below (1–6) the 1300 m elevation threshold. Flea diversity was significantly higher for sites above 1300 m than below; Wilcoxon rank sums test with chi square approximation χ = 6.55 d.f. = 1 P = 0.01).

For each host species or genus, we compared abundances between sites situated above or below 1300 m elevation. With the exception of *Mus minutoides*, which was more abundant above 1300 m (median 0.04 *M. minutoides*/trap: range: 0.03–0.04 *M. minutoides* /trap) than below (0.01 [0–0.03]; Wilcoxon rank sums test with chi square approximation χ^2^ = 5.5 d.f. = 1 P = 0.02), host abundances were similar between high and low elevation sites. In addition, host diversity was similar between sites above and below 1300 m (figure 4). Likewise, median numbers of fleas and fleas per host were similar between sites above and below 1300 m. Thus, it is unlikely that differences in plague risk, which are associated with this elevation threshold, result from differences in host or flea abundance or from differences in host diversity. By contrast, flea diversity was significantly higher for sites above 1300 m (Simpson's index of diversity median 0.73; range: 0.73–0.75) than below (0.20 [0–0.65]; Wilcoxon rank sums test with chi square approximation χ = 6.55 d.f. = 1 P = 0.01) (figures 3 and 4). Flea diversity was positively associated with elevation and elevation explained 79% of the observed variation in flea diversity (Flea diversity = 0.0009 m^−1^* elevation−0.55; F = 30.17, d.f. = 1,9, P = 0.0006).

### Associations between flea diversity and site-specific measures of temperature and rainfall

Site-specific cumulative rainfall during the month of July explained 90% of the variation observed in the flea diversity index (F = 68.40, d.f. = 1,9, P<0.0001; Flea diversity = 0.006 mm^−1^ * Cumulative rainfall in July – 0.276). Overall, rainfall amounts in July were significantly higher for high elevation sites (median 178.46 mm, rang: 162.76–193.71 mm) relative to low elevation sites (81.99 mm [63.46–132.98 mm]; χ = 6.55 d.f. = 1, P = 0.011). It should be noted that with the exception of May, October and December, rainfall within each month yielded a significant positive association with the site-specific flea diversity index. These months may represent time periods when rainfall was similarly above (during the heavy rains) or below (during the dry season) critical rainfall thresholds for sites above or below 1300 m. Flea diversity was negatively associated with the average monthly maximum temperature in the warmest month of the year (February) and this relationship explained 83% of the observed variation in site-specific flea indices. (F = 39.1, d.f. = 1,9, P = 0.0002; Flea diversity = −0.105 K-1 * Tmax (Feb)+32.53). Thus, diversity was lower in areas below 1300 m that are the hottest during the February heat. Average maximum temperature for February was significantly lower above 1300 m (301.96 K [301.05–302.80 K]; 29.8°C [28.9–30.7°C]) than below (306.34 K [304.24–308.37 K]; 34.2°C [32.1–36.2°C]).

## Discussion

Throughout a year of sampling along an elevation gradient spanning from low elevation sites situated outside of the West Nile plague focus, to higher elevation sites within the focus, we did not identify any significant differences in host abundance or diversity between sites within the defined plague focus compared with outside the focus. Likewise, numbers of host-seeking fleas, fleas recovered from hosts, and fleas per host were similar inside compared with outside of the focus, suggesting that pathogen transmission was not a simple function of contact rates between vectors and hosts. By contrast, we detected significant differences in abundance of particular flea species inside compared with outside the focus. However, it is notable that *X. cheopis*, which is arguably the most efficient vector of *Y. pestis* [31], was the predominant species outside of the focus. Therefore, lack of an efficient vector outside the focus is an unlikely cause of the absence of *Y. pestis*. Furthermore, *X. cheopis* replaces *X. brasiliensis* in the northern portion of this plague focus [32], suggesting that these species fill a similar ecological niche. This observation challenges the notion that perhaps simple species replacement could explain the observed distribution in plague risk. Instead, our data implicate higher flea diversity as a significant factor in defining plague foci and may play an important role in *Y. pestis* persistence or transmission from enzootic cycles to humans. This finding contrasts the dominant view held in the 20^th^ century and prior that host ecology drives the distribution and persistence of plague bacteria [12], [13], [14]

In general, it is believed that sufficient contact rates between hosts and vectors are required to maintain enzootic transmission of *Y. pestis* and these contact rates are often dependent on host and flea abundances [33]. With one exception (*M. minutoides*), host abundance was similar between high and low elevation sites. It is unlikely that this difference accounts for significant differences in risk because these mice harbor extremely low numbers of fleas and the diversity of fleas hosted appears to be quite low. Similar abundances were noted both inside and outside the plague focus for at least three small mammal species (*R. rattus*, *A. niloticus*, and *Mastomys spp.*) that are known to be susceptible to *Y. pestis* and believed to be important in *Y. pestis* transmission in this and other parts of Africa [32], [34], [35], [36], [37], [38], [39]. Despite similar abundances of fleas inside and outside the focus, inside the focus, flea diversity was significantly higher than outside, which suggests that increased flea diversity may be important for the maintenance of *Y. pestis* transmission cycles or may be important for transmission to humans. Having multiple species of fleas within a site that are capable of transmitting *Y. pestis* (i.e., *X. cheopis*, *X. brasiliensis*, *C. cabirus*, *S. torvus*, and *D. lypusus* [31], [39], [40], [41], [42], [43], [44]) and that infest hosts that are susceptible to plague infection could increase the likelihood of pathogen persistence during periods when the abundance of any particular host or flea species decreases below levels where persistence would be possible when only a single vector species was present [45]. In other words, the presence of multiple vector species that will infest multiple susceptible host species creates a more connected host network. It is also possible that off-host adult populations of some flea species are able to survive infected for relatively long periods in burrows or nests, thus contributing to the persistence of plague in this region. Alternatively, it is possible that increased flea diversity creates greater connections between zoonotic hosts and humans. Long-term animal-based surveillance could reveal that *Y. pestis* persists in zoonotic cycles at low elevations, most likely in the sylvatic areas outside of our sampling area, but lack of a bridging vector could result in enzootic maintenance of *Y. pestis* but an absence of human plague cases.

Host and flea abundances have been shown to vary in response to temperature or rainfall [17], [45]. Indeed, although the measures used in our study are likely attenuated by microclimatic conditions, temperature and rainfall may play an important role in defining the distribution of certain vector species. We showed that flea diversity is strongly and positively associated with rainfall and negatively associated with temperature. It is possible that wetter and cooler conditions at the higher elevation sites are more conducive to the survival of certain flea species (i.e. *X. brasiliensis*, *C. cabirus*, and *D. lypusus*). It is most likely that temperature and moisture affect the immature life stages, which exist primarily off of hosts and are presumably more sensitive to temperature and moisture fluctuations, than adults [46], [47].

Interestingly, temperature (often captured by elevation) and rainfall variables (often captured by greenness of vegetation indices) frequently emerge in spatial risk models as the best predictors of plague risk areas around the world [5], [6], [7], [9], [11], [48]. To some extent, temperature may be a predictor of plague foci because it influences the ability of fleas to survive, which may contribute to persistence. Temperature also influences the ability of *Y. pestis* to form biofilm, which might enable fleas to maintain *Y. pestis* infections [49]. Biofilm production is regulated by the hemin storage locus gene complex, and biofilm production appears to be optimized at 20°–26°C [2]. Interestingly, within our study sites, average maximum temperature for sites above 1300 m ranges from 25.3° to 26.9°C, whereas sites below 1300 m range from 28.3° to 32.7°C. However, it is important to note that adult fleas of some species typically remain on their mammalian hosts rather than spending most of their time in nests or burrows, so the temperature range experienced by these fleas is somewhat buffered from ambient temperatures [42]. In addition, the temperatures reported represent the maximum temperatures observed for these sites, and these maxima are still below the temperature at which hms proteins begin to degrade, circa 37°C [2].

Flea community composition, which also varies with elevation, could play a role in explaining the geographic distribution of plague bacteria. Plague foci are typically situated in tropical and sub-tropical latitudes and extend into warmer regions of temperate latitudes. However, within these broad limits, *Y. pestis* is usually absent in deserts and large continuous forests, and foci are commonly restricted to highlands, but absent from extremely high elevations [3]. Consistent with our observations at a very local scale that flea diversity is higher within higher elevation sites, several studies from other geographic regions that were reviewed recently [50] have noted that flea species diversity and richness are higher at higher elevation sites, but depressed in very high elevations. However, these studies did not differentiate between plague endemic and non-endemic regions. Indeed, studies of host and vector diversity along transects that span from inside to outside of plague foci are uncommon. During examination of one instance where attempts were made to compare flea diversity inside and outside of a focus in Central Java [51], we noted remarkable similarity to our findings in the West Nile region. In both regions, *Y. pestis* is restricted to higher elevation sites (above 1,000 m in Central Java). Likewise, both primary and secondary hosts are present inside and outside the Central Java focus, but flea diversity is lower below 1000 m with the proposed vectors *Stivalius cognatus* and *Neopsylla sondaica* absent or rare below this elevation [51].

The generality of our observation that flea diversity is higher in plague foci compared with outside requires further evaluation. In one instance, Laudisoit and others (2009) sought to compare host and flea communities in areas of Tanzania where plague was endemic to areas where plague was rare or absent. Similar to our study, they found no difference in host density between sites. However, in contrast to our study, they did not detect differences in any variables related to flea abundance or diversity. We argue that this may be because the sites evaluated encompassed sites where cases were rare, rather than absent, thus all sites were contained within the plague focus. Indeed, when looking at similarly defined sites within the West Nile focus (i.e., villages with or without a history of plague), host and flea diversity and abundance were similar between sites (Amatre et al. 2009).

As reviewed recently (Gage and Kosoy 2005, Eisen and Gage 2009), there has been extensive discussion, but few solid conclusions, on how *Y. pestis* is maintained during inter-epizootic periods. Many of the most common hypotheses focus on host abundance, host community composition, spatial configurations of host populations, or survival of *Y. pestis* outside of vertebrate hosts. Although survival of *Y. pestis* within infected fleas has been proposed repeatedly, the importance of flea diversity in *Y. pestis* persistence has seldom been addressed. These findings highlight the need to explicitly consider flea diversity within models of *Y. pestis* persistence.
